# Prospective Study on Factors Associated with Referral of Patients with Opioid Maintenance Therapy from Specialized Addictive Disorders Centers to Primary Care

**DOI:** 10.3390/ijerph18115749

**Published:** 2021-05-27

**Authors:** Morgane Guillou-Landreat, Philippe Levassor, Marylène Guerlais, Veronique Sebille, Caroline Victorri-Vigneau

**Affiliations:** 1 INSERM UMR 1246, SPHERE, Methods in Patient-Centered Outcomes and Health Research, Nantes and Tours Universities, 44000 Nantes, France; morgane.guillou@chu-brest.fr (M.G.-L.); veronique.sebille@univ-nantes.fr (V.S.); 2 EA 7479 SPURBO, Université Bretagne Occidentale, 29200 Brest, France; 3 HUGOPSY Network, 35000 Rennes, France; 4 CSAPA La Rose des Vents, 44600 Saint Nazaire, France; philippe.levassor@gmail.com; 5 CHU Nantes, Pharmacology Department, 44000 Nantes, France; marylene.guerlais@chu-nantes.fr

**Keywords:** opiate maintenance treatment, methadone, buprenorphine, primary care, opiate dependence

## Abstract

*Background*: One of the most important issues for opiate maintenance therapy efficacy is the involvement of primary care physicians (PCPs) in opiate use disorder treatment, especially after referral from specialized units. This study aimed to analyze the progress of subjects in a specialized center and after referral to PCPs. *Methods*: This study was an observational prospective study. Recruitment took place in a specialized addictive disorder center in western France. All patients were evaluated (sociodemographical data, severity of substance use disorders through the TMSP scale, the quality of life through the TEAQV scale) by physicians during the 5-year-follow up of the study. Analysis focused on four main times during follow-up: entry/last visit into specialized care and into primary care. *Results*: 113 patients were included in this study; 93% were receiving methadone and 7% buprenorphine. Ninety (90) were referred to primary care. In primary care follow-up, the probability of the lowest severity score for substance use disorders remained stable over time. *Conclusions*: In daily practice, a center specialized in addictive disorders referred OMT management to PCPs for a majority of patients, and benefits regarding substance use disorders severity and quality of life remained stable after referral. Our results need to be confirmed.

## 1. Introduction

Opioid use disorders (OUD), whether related to illicit or non-medical drug use, are a major public health issue worldwide. About 15,479,000 people around the world are affected by opioid dependence [1]. Over more than 20 years, opioid maintenance therapies (OMT) have been approved in a growing number of countries and have been used in the therapeutic management of related medical, social and psychological conditions. OMT has amply proved its effectiveness and its value in terms of public health in the reduction of morbidity and mortality [2,3,4,5]. OMT prescription has different objectives: to avoid opioid withdrawal symptoms, to block the effects of illicit opioids, to reduce opioid craving, to stop or reduce the use of illicit opioids, to prevent relapse, to stop drug injections so as to reduce risks of infection, and finally to promote and facilitate patient commitment to recovery-oriented activities including psychosocial interventions [6,7,8]. Recommendations underline the need to maintain OMT over a long period of time [6,9]. This is a way of reducing the high risk of mortality due to opioid overdose [10], estimated at 30 deaths/1000 person-years in the 4 weeks after OMT cessation [11]. Nevertheless, most individuals with an addiction do not receive treatment [12,13,14], and the accessibility of OMT and the legislation vary considerably from one country to another.

France differs from most other European countries in two respects: firstly France is among the European countries with the largest numbers of patients on OMT in relation to its population aged 15 to 64; secondly prescriptions of buprenorphine (BHD) are markedly dominant (15). This situation results from specific legislation in France concerning OMT.

Methadone was marketed in 1995 and buprenorphine in 1996, and they are both available in France as part of a global therapeutic strategy. They are prescribed according to strict guidelines. Buprenorphine, which is a partial mu-opioid agonist, can be prescribed and initiated (for a maximum duration of 28 days) by primary care physicians (PCPs) in private practice, with dispensation through community pharmacies [15]. Methadone, which is a pure mu-opioid agonist listed as a narcotic, is less accessible. Its primary prescription is restricted to physicians operating in units specialized in addictive disorders or hospitals. After a period of stabilization, follow-up and prescription can be carried out by any physician [3,9,16].

This “French model” [17] contrasts with other countries worldwide where methadone is predominantly used [18]. Collaboration between PCPs and community pharmacies guarantees the performance of this model [19]. As a consequence, the involvement of PCPs in OUD management, especially after referral from specialized units, is a major issue [20]. For a long time in France, the management of more than three-quarters of patients with an OUD was ensured by 20% of French PCPs [16], but recently, some authors have underlined changes in OMT prescribing habits among PCPs: a decrease in buprenorphine prescription and an increase in the number of PCPs prescribing methadone, which argues for an involvement of PCPs in referral from addiction specialized centers or hospitals [21].

But surprisingly, although there has been interest and studies on OMT treatment, either in specialized centers [22,23,24], or in general practice [25,26,27,28], to our knowledge, no study has analyzed patient trajectories from entry into a specialized center to follow-up in general practice. None have focused on the long-term progression of patients through the care system from specialized centers to primary care.

## 2. Materials and Methods

### 2.1. Aim and Design and Setting of the Study

#### 2.1.1. Aim

Our study aimed to prospectively analyze whether the benefits of specialized treatment, of subjects following OMT for opioid use disorders, with respect to the severity of the addictive disorders were maintained after referral from the specialized center to primary care. Our secondary objectives were to characterize the evolution of quality of life, and factors associated with the transition from medical centers specialized in addictive disorders to general practice.

#### 2.1.2. Design

This study was an observational prospective study, conducted by the Nantes University Hospital Addictovigilance department. The study was funded by a grant from the French national Mission Interministérielle de Lutte contre les Drogues Et les Conduites Addictives (MILDECA) and was monitored by a multidisciplinary steering committee made up of pharmacologists, psychiatrists specialized in addiction, biostatisticians and PCPs.

This study was approved by an ethics commitee (CPP), the Comité Consultatif sur le Traitement de l’Information en matière de Recherche dans le domaine de la Santé (CCTIRS) and the French Commission Nationale de l’Information et des Libertés (CNIL). All participants provided written informed consent in accordance with the Declaration of Helsinki.

#### 2.1.3. Setting

Patient recruitment took place in a specialized addictive disorder center (CSAPA) in western France. In this center, patients seeking treatment for an OUD have a multidisciplinary follow-up including medical, psychological and social care. These specific missions are defined for all the French addictive disorder centers (CSAPA) by a national decree [29]. OMT is then prescribed, after an individualized evaluation, and dispensed daily in the center.

The primary care model was a physician-centered model: a single physician or group of physicians working together to provide patient-centered OUD treatment without major structural support from other types of provider or disciplines. The physician independently counseled and treated the patient. Specialized addictive disorder center transferred medical data to the PCP at the moment of the referral through a medical mail: information regarding addictive disorders, medical and psychiatric history and regarding treatment (OMT and associated treatment). Once the patient was referred in primary care, the PCP was the manager of the patient′s situation. Data was not transferred from PCP to specialized addiction centers. PCPs were required to meet patients for OMT renewal at least every 14 days for MTD syrup, and every 28 days for MTD tablets or buprenorphine. The PCPs worked with community pharmacies for dispensation (weekly, apart from exceptions).

### 2.2. Characteristics of Participants and Materials

#### 2.2.1. Participants

To be eligible, patients were to be over 18, and seeking treatment for an opioid use disorder in a center specialized in addictive disorders. Informed consent was obtained from all subjects involved in the study. Patients who were not fluent in French were excluded from the study.

All patients were evaluated by physicians (i) first at entry into the specialized units, then 2 months later, then every 6 months and also at the end of the specialized unit follow-up; then (ii) after referral to primary care, at entry and then every 6 months over a 5-year period.

#### 2.2.2. Data Collected

Follow up was individualized according the moment of the entry of each patient in specialized care, and the moment they were referred. All the data were collected at each visit from the inclusion of each individual to the end of his personal follow up. All patients were evaluated by physicians (i) first at their entry into the specialized unit, 2 months later, then every 6 months and also at the end of the specialized unit follow-up; then (ii) after their referral to FPs, at entry and then every 6 months over a 5-year period. Only the data concerning entry into specialized unit, end of the follow up in the specialized unit, entry in primary care and end of follow up in primary care were used for this study.

#### 2.2.3. Sociodemographic and Medical Data

Data collected included sociodemographic data (age, gender, living with a partner, children, income), medical data (physical health problems and psychiatric issues identified in a clinical medical interview); knowledge of serological status: (HIV, HCV, HBV self-reported by patients, precautions taken by patients for risk reduction of virus transmission, self-reported), psychoactive substance consumption.

#### 2.2.4. The TMSP

The severity of addictive disorders was evaluated using the TMSP [30]. This multidimensional scale measures the severity of substance use disorders according to four dimensions: Substance use (T), Medical score (M), Social Score (S), Psychiatric score (P). Each dimension is expressed in 3 levels of severity, and the overall score determines a global severity stage (from Stage A (the least severe) to stage D (extremely severe). (Appendix A).

This tool, unlike other validated severity scales, was designed for use in primary care [30]. It was chosen by the multidisciplinary steering committee because it is a simple tool, and easily repeatable in routine care.

#### 2.2.5. The TEAQV

In this study, the method required an evaluation in routine practice, so the multidisciplinary steering committee decided to use the simple French tool known as the TEAQV(Assisted Evaluation of Quality of life), to explore quality of life [31].

This instrument is a two-part. 7 point-scale (0 = extremely bad; 6 = excellent), self-rated quantitative evaluation of quality of life at different time points in 4 areas (physical and psychological well-being, family relationships, professional activity). The first part is a one-time retrospective lifetime evaluation whereas the second part is a current state evaluation that can be prospectively repeated. This instrument is administered in 5 to 10 min. The self-assessment of the TEAQV scale was assisted: the interviewer told the patient the time frame (Entry and last visit in specialized care, entry and last visit in primary care). For the following evaluations the subject completed the initial values with a new line corresponding to the period that has just elapsed, and so on for each evaluation provided for in the protocol.

Strada et al. in a systematic review identified 16 tools for evaluation of quality of life among opioid-dependent patients [32]. They underline that it is especially recommended to use questionnaires that are practical to use in routine patient care, in order to further bridge the gap between research and practice [32]. It was thus the main criteria of choice for the TEAQV in this study.

### 2.3. Outcomes

The primary outcome was the change in severity of the addictive disorders among patients following OMT from arrival in the specialized center to referral to primary care and continuing until the end of follow-up. The severity of addictive disorders was measured using the TMSP score, where Stage A is the least severe category. Change in the severity of the addictive disorders was measured by the change in the proportion of patients in stage A.

The secondary outcomes were time from entry in specialized care to primary care referral and identification of the covariates associated with the referral, and the change in quality of life (measured through the TEAQV) from entry in the specialized center to referral to primary care and continuing until the end of follow-up. We focused on four main transition times during follow-up: entry into specialized care and last visit, entry into primary care and last visit.

### 2.4. Statistical Analysis

Continuous data are expressed as means (±SD) and categorical data as numbers and percentages. Changes in the proportions of patients in stage A from inclusion in the specialized center to referral to primary care and on to the end of follow-up was assessed using generalized estimating equations (GEE). Model fit was assessed using quasi-likelihood under the Independence Criterion (QIC) [33]; a lower QIC value indicates a better model fit. Standard errors for the parameters were obtained using the empirical so-called “sandwich” estimator. The same analysis was performed to assess changes in QoL domains, using the proportion of patients for whom QoL domains were rated at least as “quite good”. These analyses were performed on two populations: 1/the whole sample of patients, whether referred or not to primary care and 2/restricted to patients who were referred to primary care.

The survival distribution of general practice referral was estimated using Kaplan–Meier estimates. The covariates associated with this distribution were selected using backward selection using Cox regression. A *p* value of <0.05 was considered statistically significant. Statistical analyses were performed using SAS statistical software (SAS 9.4, SAS Institute, Cary, NC, USA).

## 3. Results

### 3.1. Characteristics of the Patients

113 patients were included in this study: 93% had been prescribed methadone and 7% had been prescribed buprenorphine. Regarding OMT, at entry 54.87% (*n* = 62) of the sample reported having obtained OMT in other ways than through medical prescription, mainly on the streets for 54.0% (*n* = 61), 0.88% (*n* = 1) had already stolen a medical prescription, and 1.77% (*n* = 2) had falsified a medical prescription to obtain OMT. All the participants (100%, *n* = 113) reported current consumption of psychoactive substances (excluding prescribed OMT).

**Figure 1:**
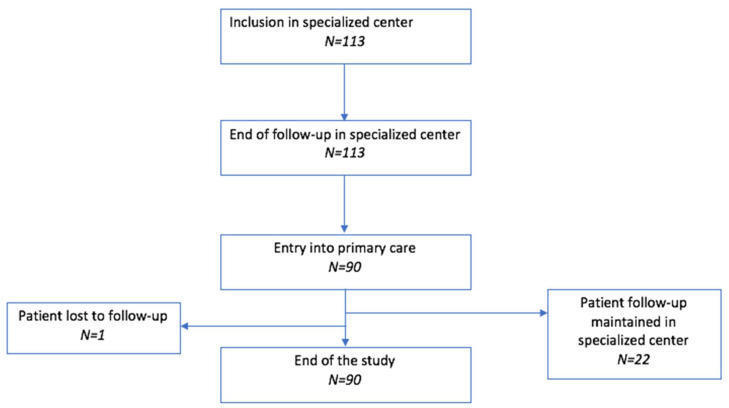
Study flowchart. (*N* = Number of patients).

Twenty-three patients remained and were followed up in the center specialized in addictive disorders, and ninety patients were referred to primary care (see flowchart—Figure 1). The socio-demographic, medical data and damage associated with OUD at the inclusion in the specialized center are presented in Table 1.

Figure 1 provides the flowchart of the study, with the numbers of patients at the four transition times in the overall follow-up (entry into specialized care and last visit, entry into primary care and last visit).

The descriptive analysis of the severity of addictive disorders according the TMSP score and quality of life according the TEAQV scale are reported in Table 2 at the four transition times in the overall follow-up (entry into specialized care and last visit, entry into primary care and last visit).

### 3.2. Primary Outcome

#### 3.2.1. Whole Sample of Patients, Whether Referred or Not to Primary Care

**Figure 2:**
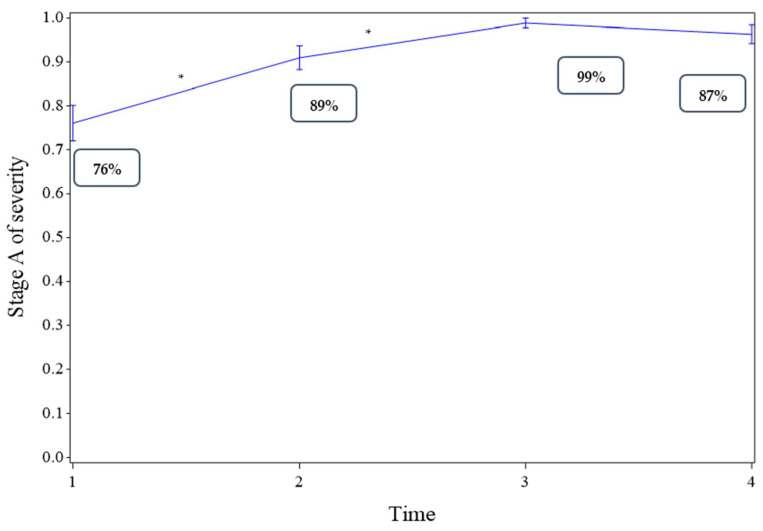
Changes in the proportion of patients in the least severe category (Stage A) for the severity of addictive disorders estimated using generalized estimating equations (GEE). A significant time effect is indicated by “*”. Time 1: at entry into the specialized center (inclusion); Time 2: last visit to the specialized center; Time 3: at entry into primary care; Time 4: last visit to primary care.

The changes in the proportions of patients in Stage A, from inclusion in the specialized center to primary care referral and up to the end of follow-up, are shown in Figure 2. The proportion of patients in Stage A increased significantly between the inclusion of patients (Time 1, Figure 2) and the last visit in the specialized center (Time 2, Figure 2, *p* < 0.001). It also increased between Time 2 and entry into primary care (Time 3, Figure 2, *p* = 0.022) and remained stable afterwards until the last visit to primary care (Time 3 versus Time 4, Figure 2, *p* = 0.258).

#### 3.2.2. Restricted to Patients Who Were Referred to Primary Care

The proportion of patients in Stage A increased significantly between the inclusion of patients (Time 1, see supplementary material Figure S1 and the last visit in the specialized center (Time 2, Figure S1, *p* < 0.001). It remained stable afterwards until the last visit to primary care (Time 2 versus Time 3, Figure S1, *p* = 0.520; Time 3 versus Time 4, Figure S1, *p* = 0.279).

### 3.3. Secondary Outcomes

#### 3.3.1. Time to Referral to General Practice

**Figure 3:**
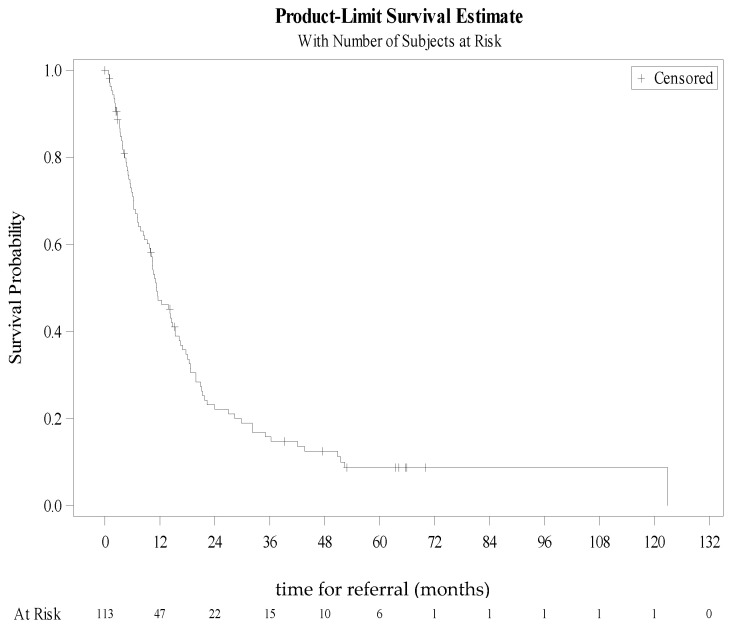
Kaplan-Meier curve estimating time to referral to primary care.

The Kaplan-Meier curve estimates of time to referral to general practice are shown in Figure 3.

Among the 113 patients, 91 were referred to general practice, and one patient was lost to follow-up between specialized care and primary care, so that 90 patients were included in primary care follow-up. The median time to general practice referral was 11.3 months, i.e., about a year. Variables associated with the time to referral are presented in Table 3.

#### 3.3.2. Patient Characteristics Associated with Primary Care Referral

Having a partner, having children, or being in the least severe category (Stage A) for addiction severity significantly increased the chances of being referred to primary care. More specifically, patients in Stage A who have not yet been referred to primary care have more than twice the likelihood of being referred to primary care compared to patients in Stages B, C, or D (hazard ratio (HR) = 2.412, 95% confidence interval (CI) [1.284–4.528]). In contrast, knowledge of serological status was associated with about a 40% lower likelihood of being referred to primary care (HR = 0.601, 95% CI [0.392–0.922]).

### 3.4. Changes in Quality of Life (QoL)

#### 3.4.1. Whole Sample of Patients, Whether Referred or Not to Primary Care

**Figure 4:**
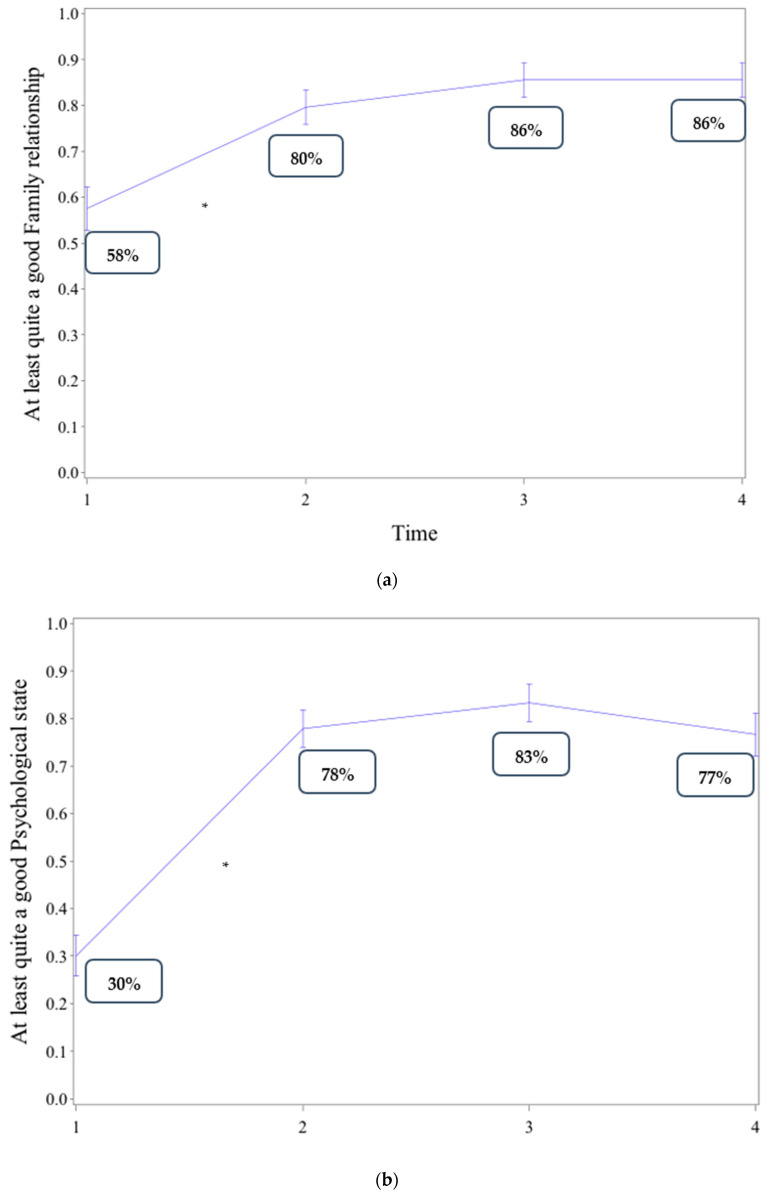

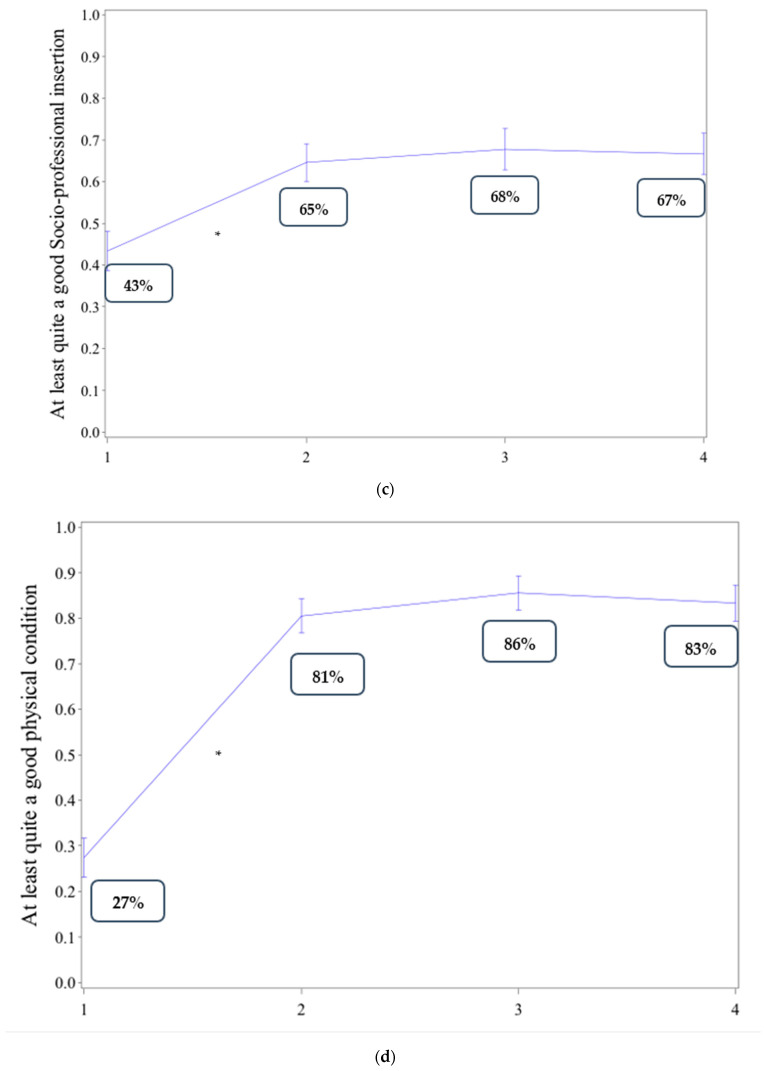
(**a**) Change in the proportion of patients whose QoL ratings for family relationship were at least “quite good”, at different times. (**b**) Change in the proportion of patients whose QoL ratings for psychological state were at least “quite good”, at different times. (**c**) Change in the proportion of patients whose QoL ratings for socio-professional insertion were at least “quite good”, at different times. (**d**). Change in the proportion of patients whose QoL ratings for physical condition were at least “quite good”, at different times.

Changes in the proportion of patients for whom QoL domains were rated at least as “quite good”, from inclusion in the specialized center to primary care referral and up to the end of follow-up, are shown in Figure 4. For all QoL domains, the proportion of patients whose QoL ratings were at least “quite good” significantly increased between the inclusion of patients (Time 1, Figure 4) and the last visit to the specialized center (Time 2, Figure 4, *p* < 0.001 for all QoL domains). However, it remained stable afterwards up to the last visit to primary care (Time 4, Figure 4, *p* > 0.25 for all QoL domains).

#### 3.4.2. Restricted to Patients Who Were Referred to Primary Care

The same pattern of change in QoL domains was observed for patients who were referred to primary care (see Supplementary Material Figure S2a–d).

## 4. Discussion

This prospective study in daily practice analyzed the evolution of the level of severity of addictive disorders among 113 subjects following OMT for an OUD after referral from a specialized center to primary care.

The main result was that the benefits of OUD management, combining OMT and medical and psychosocial care, were significant during the follow-up in specialized center and were maintained thereafter in primary care, in terms of both addictive disorder severity and quality of life. The proportion of patients in the least severe category for addictive disorders increased significantly from the inclusion of patients to the last visit in the specialized center. These results could reflect the short term impact of medical and psychosocial treatment of OUD. OMT have a clear positive impact, when it is combined with psychosocial interventions, as is the case in specialized centers [34,35,36,37,38]. But this study also showed that these benefits concerning addictive disorders severity remained stable, after referral on primary care and afterwards up to the last visit to primary care. These results are really important, as two-thirds of OMT are prescribed by primary care physicians in France [39]. PCP need to be involved in patient follow-up, to improve access to OMT, in patient-centred program [9].

Regarding the evolution of quality of life, an improvement was also identified in all domains of QoL and it was maintained after referral: the proportion of patients reporting at least “quite good” quality of life increased between entry into specialized center and primary care and remained stable until the last visit to primary care. OMT benefits concerning reduction of opioid craving, improvement of treatment retention, reduction of illicit opioid use, and increasing overall survival are well- known [40]. Benefits on quality of life are less explored in the literature, but our study showed that in a patient centered approach, the quality of life remained stable in a primary care follow up.

A large majority of patients in this study (79%) were referred to primary care during the 5 years of the study, the median duration of follow-up in the center specialized in addictive disorders was 1.5 years, and 50% were referred within 280 days of entry into the center specialized in addictive disorders. This suggests that, as underlined by Dupouy et al., GPs are increasingly implicated in management of patients for the monitoring of these treatments after their initial care in addiction specialisation centres [21].

Referral to primary care was correlated with the global evaluation of severity of the addictive disorders, patients in the lowest severity category (stage A) who had not yet been referred to primary care had more than twice the likelihood of primary care referral compared to patients in Stages B, C, or D (HR = 2.412). One hypothesis is that specialized addictive disorder centers, where patients have access to more psychological, medical and social support, prefer to refer the lowest severity profiles to primary care and to maintain the most severe patients in their facility for treatment. The difference in the proportion of patients in the lowest severity category on the TMSP between the last visit to specialized care and the first visit to primary care is consistent with this hypothesis, since the most severe patients were not included in the analysis of the first primary care visit. Indeed, the treatment of co-existing psychiatric disorders is important and, whenever possible, it should be provided by individuals or teams with expertise in the management of dual diagnoses [9]. The treatment of medical comorbidities, such as infectious diseases, is also part of the global approach in the treatment of OUD in specialized centers [9]. In our study, knowledge of serological status was associated with about a 40% lower likelihood of being referred to primary care. It can be supposed that patients aware of their serological status could more frequently be involved in risky behaviors. This could correspond to the recommended Integrated, multidisciplinary care models in OUD, integrating patients at risk for infectious comorbidities, an approach that is critical in preventing the continuing spread of HIV and HCV [41].

Environmental variables also influenced patient referral, and patients with a partner and with children were more likely to be referred than those without a partner or children. These two variables could reflect stable housing conditions, and studies have shown that individuals with an OUD and under OMT living with a partner or with family members or friends are less likely to use heroin during treatment and show a higher treatment compliance [42,43]. Another hypothesis is that referral could be motivated by practical considerations, as primary care follow-up is less demanding and more compatible with a family life. OMT management in addictive disorder centers can be restrictive, while office-based settings and outpatient programs offer greater anonymity and are less stigmatizing, more particularly for socially integrated patients. Even if primary care settings have less expertise in addiction medicine, they have more expertise concerning patient history and a global view of the patient’s health [21,44]. These environmental variables were also associated with a shorter time before referral to primary care. Better social integration (people with children and a stable family) seemed to be a variable that care providers in the center specialized in addictive disorders identified and appeared to facilitate a more rapid referral in primary care. It could also reflect patient motivations and expectations. A study in Ireland interviewed clients of a methadone maintenance program and they showed that a structured living environment and familial incentive, mainly family members, particularly children or younger siblings, were positive factors in patient trajectories [45].

The moment of primary care referral was also influenced by global severity: severe patients, combining familial problems, medical morbidities and higher levels of severity of addictive disorders, were significantly less rapidly referred to primary care in this study. Having family problems, being in a high severity category for addictive disorders (Stage B or C) and knowledge of serological status (HIV, HVC, HVB) were associated with later referral.

### 4.1. Implications

In a patient-centered approach, the development of OUD management in primary care, in collaboration with specialized centers, is necessary. In this study we showed that in daily practice, a center specialized in addictive disorders referred OMT management to primary care for a majority of their patients. A prioritization of interventions according to profiles seemed to emerge: the least severe clinical profiles, more socially integrated and stable (with children, living with a partner) were more frequently and more rapidly referred, while the most severe profiles, combining social and familial problems, and medical disorders (knowledge of serological status- (HIV-HCV or HBV)) were maintained in a specialized center. Maintaining patients in a center specialized in addictive disorders could reinforce stigmatization and some studies report that a negative atmosphere in the treatment setting constitutes a barrier to the instatement of OMT [46,47], probably more particularly for those who are well-integrated. In a pragmatic randomized trial on methadone instatement in primary care in France, primary care appeared to be more attractive and acceptable than specialized care [48].

Despite being less able to provide psychological or social support or having less expertise in addiction medicine, PCPs have a wide expertise on the patient’ history and a global view of his/her health [21]. In our study, the benefits of OUD management and OMT were maintained, in terms of the proportion of patients in the least severe addictive disorder category (Stage A) and also in terms of quality of life. As shown in a previous study, patient satisfaction rates between OUD management in primary care and management in specialized care are statistically significantly higher for primary care, and a meta-analysis of the effect of the treatment setting on treatment compliance found 86% in primary care versus 67% in a specialty clinic [49]. Opioid maintenance therapy management requires care centered on family practice [27].

However, a considerable number of barriers to OMT prescription still remain (3, 21) [50]. OMT is considered by a large majority of patients and also by physicians and pharmacists as a type of treatment that is unlike other treatments (21). Many physicians feel ill-prepared to prescribe OMT (22) or feel they lack sufficient training and experience (3). Only a minority of early-career family physicians report having received adequate preparation to provide buprenorphine treatment during their residency [3]. Even among trained physicians, the majority do not prescribe because of logistic barriers (23). Finally, there is another barrier which concerns specialized addiction centers and uncertainties about progress among patients after referral to primary care. Our results need to be confirmed, and a randomized controlled trial could compare the evolution of patients with an OMT in specialized centers with that in primary care.

### 4.2. Strengths and Limitations

This prospective study enabled follow-up of 90 patients from specialized centers to primary care, in daily routine practice, and this is the main strength of the study. These prospective data for a significant sample of 113 patients, recruited in a daily clinical practice, with a follow up from specialized care to primary care are original, in comparison to existing literature on the topic, and important face to the importance of this topic [44,49].

The tools selected to evaluate the severity of the addictive disorder and quality of life were not classic for clinical research on the topic, and data regarding validity and reliability of these tools were missing in literature but for this pragmatic study, they combined several qualities: easy to use in routine practice for any physicians or patients, and easily repeatable.

In addition, this was an observational study which can be considered a limitation. There might also have been a selection bias, as inclusions were conducted in a single specialized center. This selection bias could have influenced the severity of addictive disorders of the sample and it also limits the generalisation of the results.

## 5. Conclusions

In this study we showed that in daily practice, a center specialized in addictive disorders referred OMT management to primary care for a majority of their patients, and that benefits regarding substance use disorders severity and quality of life remained stable after referral. A prioritization of interventions according to profiles seemed to emerge: the least severe clinical profiles, more socially integrated and stable (with children, living with a partner) were more frequently and more rapidly referred. Our results need to be confirmed, and a randomized controlled trial could compare the evolution of patients with an OMT in specialized centers with that in primary care.

## Figures and Tables

**Table 1:** Description of sociodemographic data at inclusion in the specialized center.

Variables	*n* = 113	%
Sociodemographic Variables		
Age, years, mean (SD)	28.6 (6.2)	
Gender, Male	85	75.2
With social support	111	98.23
Living with a partner	48	42.48
Children	38	33.63
Drug users among persons around	95	84.07
Drug users among spouses	20	17.70
Drug users among friends	92	81.42
Stable housing	86	76.11
Professional activity	66	58.41
Regular income	68	60.18
Comorbidities		
Psychiatric comorbidities	47	41.59
Medical comorbidities	9	7.96
Knowledge of serological status	55	48.67
HIV +	1	0.88
HCV+	5	4.42
Patients not taking precautions to reduce risks of virus transmission	36	31.86
Damage associated with Opioid Use disorders		
Familial damage	43	38.05
Professional damage	53	46.90
Medical damage	26	23.01
Legal damage	29	25.66

**Table 2:** Severity and quality of life at the four main transition times in the overall follow-up (entry into specialized care and last visit, entry into primary care and last visit).

Variables	At Inclusion in the Specialized Center *n =* 113 % (*n*)	Last Visit in the Specialized Center *n =* 113 % (*n*)	First Visit in Primary Care *n =* 90 % (*n*)	Last Visit in Primary Care *n =* 90 % (*n*)
**TMSP Scale**				
S- Socio-personal axis				
Satisfactory social, professional, familial situation	49.6 (56)	65.5 (74)	83.3 (75)	77.8 (70)
Social or family problem	40.7 (46)	30.1 (34)	16.7 (15)	14.4 (13)
Daily drifting and major legal risks	9.7 (11)	3.5 (4)	0.0 (0)	1.1 (1)
Missing data	0.0 (0)	0.9 (1)	0.0 (0)	6.7 (6)
M- Medical follow-up axis				
Regular follow-up and treatment compliance	49.6 (56)	82.3 (93)	95.6 (86)	86.7 (78)
Irregular follow-up and treatment compliance issues	44.3 (50)	15.0 (17)	3.3 (3)	4.4 (4)
No follow-up	6.2 (7)	1.8 (2)	1.1 (1)	1.1 (1)
Missing data	0.0 (0)	0.9 (1)	0.0 (0)	7.8 (7)
P- Psychiatric approach axis				
No significant personality disorder	58.4 (66)	74.3 (84)	81.1 (73)	74.4 (67)
Moderate personality disorders	32.7 (37)	18.6 (21)	18.9 (17)	15.6 (14)
Major personality disorders requiring specialized environment support	8.9 (10)	6.2 (7)	0.0 (0)	2.2 (2)
Missing data	0.0 (0)	0.9 (1)	0.0 (0)	7.8 (7)
T- Substance addiction axis				
1 point	49.6 (56)	85.8 (97)	95.6 (86)	76.7 (69)
2 points	44.3 (50)	10.6 (12)	4.4 (4)	11.1 (10)
5 points	6.2 (7)	2.7 (3)	0.0 (0)	4.4 (4)
Missing data	0.0 (0)	0.9 (1)	0.0 (0)	7.8 (7)
Stages of severity				
Stage A	76.1 (86)	89.4 (101)	98.9 (89)	86.7 (78)
Stage B	15.0 (17)	4.4 (5)	1.1 (1)	1.1 (1)
Stage C	6.2 (7)	3.5 (4)	0.0 (0)	1.1 (1)
Stage D	2.7 (3)	0.9 (1)	0.0 (0)	1.1 (1)
Missing data	0.0 (0)	1.8 (2)	0.0 (0)	10.0 (9)
**TEAQV Scale**				
Physical condition				
Extremely bad	1.8 (2)	1.8 (2)	1.1 (1)	1.1 (1)
Very bad	10.6 (12)	1.8 (2)	1.1 (1)	0.0 (0)
Quite bad	28.3 (32)	6.2 (7)	1.1 (1)	4.4 (4)
Medium	31.9 (36)	9.7 (11)	11.1 (10)	11.1 (10)
Quite good	20.4 (23)	36.3 (41)	31.1 (28)	34.4 (31)
Very good	6.2 (7)	38.1 (43)	43.3 (39)	34.4 (31)
Excellent	0.9 (1)	6.2 (7)	11.1 (10)	14.4 (13)
Psychological state				
Extremely bad	4.4 (5)	0.9 (1)	1.1 (1)	1.1 (1)
Very bad	14.2 (16)	3.5 (4)	0.0 (0)	0.0 (0)
Quite bad	23.9 (27)	7.1 (8)	1.1 (1)	7.8 (7)
Medium	27.4 (31)	10.6 (12)	14.4 (13)	14.4 (13)
Quite good	22.1 (25)	33.6 (38)	35.6 (32)	38.9 (35)
Very good	7.1 (8)	39.8 (45)	40.0 (36)	28.9 (26)
Excellent	0.9 (1)	4.4 (5)	7.8 (7)	8.9 (8)
Socio-professional integration				
Extremely bad	11.5 (13)	3.5 (4)	2.2 (2)	3.3 (3)
Very bad	3.5 (4)	5.3 (6)	5.6 (5)	6.7 (6)
Quite bad	16.8 (19)	9.7 (11)	3.3 (3)	12.2 (11)
Medium	24.8 (28)	16.8 (19)	21.1 (19)	11.1 (10)
Quite good	23.9 (27)	25.7 (29)	11.1 (10)	16.7 (15)
Very good	15.0 (17)	28.3 (32)	28.9 (26)	37.8 (34)
Excellent	4.4 (5)	10.6 (12)	27.8 (25)	12.2 (11)
Family relationships				
Extremely bad	8.9 (10)	4.4 (5)	1.1 (1)	2.2 (2)
Very bad	4.4 (5)	0.0 (0)	1.1 (1)	1.1 (1)
Quite bad	10.6 (12)	2.7 (3)	1.1 (1)	1.1 (1)
Medium	18.6 (21)	13.3 (15)	11.1 (10)	10.0 (9)
Quite good	17.7(20)	22.1 (25)	17.8 (16)	17.8 (16)
Very good	31.0 (35)	42.5 (48)	42.2 (38)	46.7 (42)
Excellent	8.9 (10)	15.0 (17)	25.6 (23)	21.1 (19)

Missing data are indicated where appropriate.

**Table 3:** Variables associated with the time to referral to primary care.

Variables Associated with Time to Referral to PCPs			
Variable	Hazard Ratio	CI 95%	*p*-Value
Having a partner, yes vs. no	1.755	[1.103–2.790]	0.018
Having children, yes vs. no	1.836	[1.164–2.895]	0.009
Stage of severity A vs. B, C, or D	2.412	[1.284–4.528]	0.006
Knowledge of serological status, yes vs. no	0.601	[0.392–0.922]	0.020

## Data Availability

The datasets used and/or analysed during the current study are available from the corresponding author on reasonable request.

## Supplementary Materials

The following are available online at https://www.mdpi.com/article/10.3390/ijerph18115749/s1, Figure S1: Change in the proportion of patients who were referred to primary care in the less severe stage (stage A) with regards to severity of addictive disorders, Figure S2: Change in the proportion of patients who were referred to primary care and whose QoL ratings for (a) Family relationship (b) psychological state (c) socioprofessional insertion (d) physical.

## Appendix A

### The TMSP (Lowenstein & Gourarier)

The severity of addictive disorders was evaluated using the TMSP [30]. This multidimensional scale measures the severity of substance use disorders according to 4 dimensions. Each dimension is expressed in 3 levels of severity.

1. The first dimension concerns **substance use “T** “(type of substance, route of administration of the substance, number of substances consumed, and environment with or without substance use).
  - 1 POINT: a single substance use by nasal route; little or no use of benzodiazepines, amphetamines/barbiturates or cocaine; no alcoholism; non-addicted partner; no drug dealing or addiction-related delinquency.
  - 2 POINTS: intravenous heroin addiction or poly-drug addiction (chronic drug intoxication; moderate alcohol use disorder; frequent use of cocaine, crack or amphetamines) or drug-addicted or heroin-addicted partner > 10 years or dealing or delinquency fostered by addiction.
  - 5 POINTS: polydrug addiction by the intravenous route with major de-socialisation, history or current risk of major medical complications (overdose, infections, road accidents and major trauma) or repeated legal complications (incarceration); or associated severe alcohol use disorder.
2. The second dimension concerns the **medical score “M”** (medical follow-up, compliance with treatment).
  - 1 POINT: regular medical follow-up and compliance with prescribed treatments
  - 2 POINTS: irregular medical follow-up and difficulties in treatment compliance.
  - 5 POINTS: no medical follow-up (no knowledge of serological status; no vaccinations to date; no prophylactic treatment etc.)
3. The third dimension concerns the **social score “S”** (social, workplace and familial situation).
  - 1 POINT: social, professional and family situation not worrying or satisfactory
  - 2 POINTS: social problems (no personal accommodation; papers not up to date-Social Security health insurance, 100% reimbursement status, RMI (minimum living allowance), residence permit, etc., pending lawsuits or convictions not followed up; no personal and legal resources or major debts) or family problems (loss of contact with parents and/or children)-
  - POINTS: daily drifting (homeless or squatting) with complete marginalisation and situation of abandonment, and major legal risks.
4. The fourth dimension concerns **the psychiatric score “P”** (psychiatric disorders).
  - 1 POINT: no significant personality disorder
  - 2 POINTS: moderate or controlled personality disorders, not decompensated (no history of suicide attempt, acting out or violence—in the last 6 months).
  - 5 POINTS: significant personality disorders (history of suicide attempt, violent acts or repeated psychiatric hospitalizations) requiring specialist care.

The overall score ranges from 4 to 20 and it enables classification of patients in terms of severity of addiction expressed in 4 categories (A, B, C et D):

- **Stage A** (the least severe)—Total score < 8 (4, 5, 6 and 7 points = no item scored 5 in any of the diagnostic dimensions).
- **Stage B** (severe)—Total score between 8 and 11 (8, 9, 10 and 11 points = one item scored 5 in one dimension or, for a total score of 8, 4 items rated 2).
- **Stage C** (very severe)—Total score between 12 and 16 (12, 13 or 14 points = 2 items scored 5 in the diagnostic dimensions.
- **Stage D** (extremely severe)—Total score 16 or more (16, 17 or 20 points = three or four items scored 5 in the diagnostic dimensions).

This tool, unlike other validated severity scales, was designed for use in primary care [30]. It was chosen by the multidisciplinary steering committee because it is a simple tool, and easily repeatable in routine care.
