# Supplementary material for: Prospective Study on Factors Associated with Referral of Patients with Opioid Maintenance Therapy from Specialized Addictive Disorders Centers to Primary Care
Source: Int J Environ Res Public Health. 2021 May 27;18(11):5749. doi: 10.3390/ijerph18115749 (PMC8198158; doi:10.3390/ijerph18115749)
Supplement: Supplementary file 1 [file ijerph-18-05749-s001.zip › ijerph-1116412-SI.pdf]

SUPPLEMENTARY MATERIAL

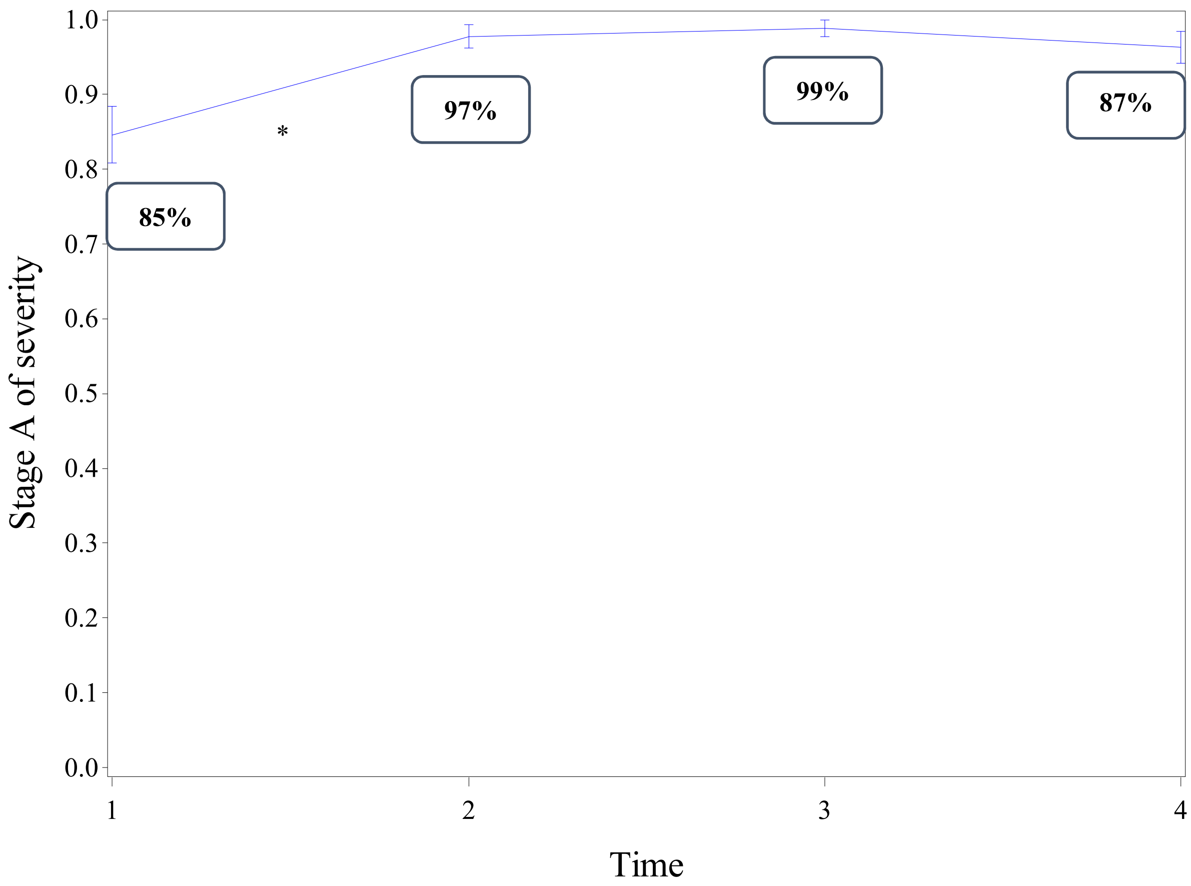

Figure S2. Change in the proportion of patients who were referred to primary care in the less severe stage (stage A) with regards to severity of addictive disorders estimated using generalized estimating equations (GEE). Significant time effect indicated by "*". Time 1: at entry in the specialized center (inclusion); Time 2: last visit in the specialized center; Time 3: at entry in primary care; Time 4: last visit in primary care.

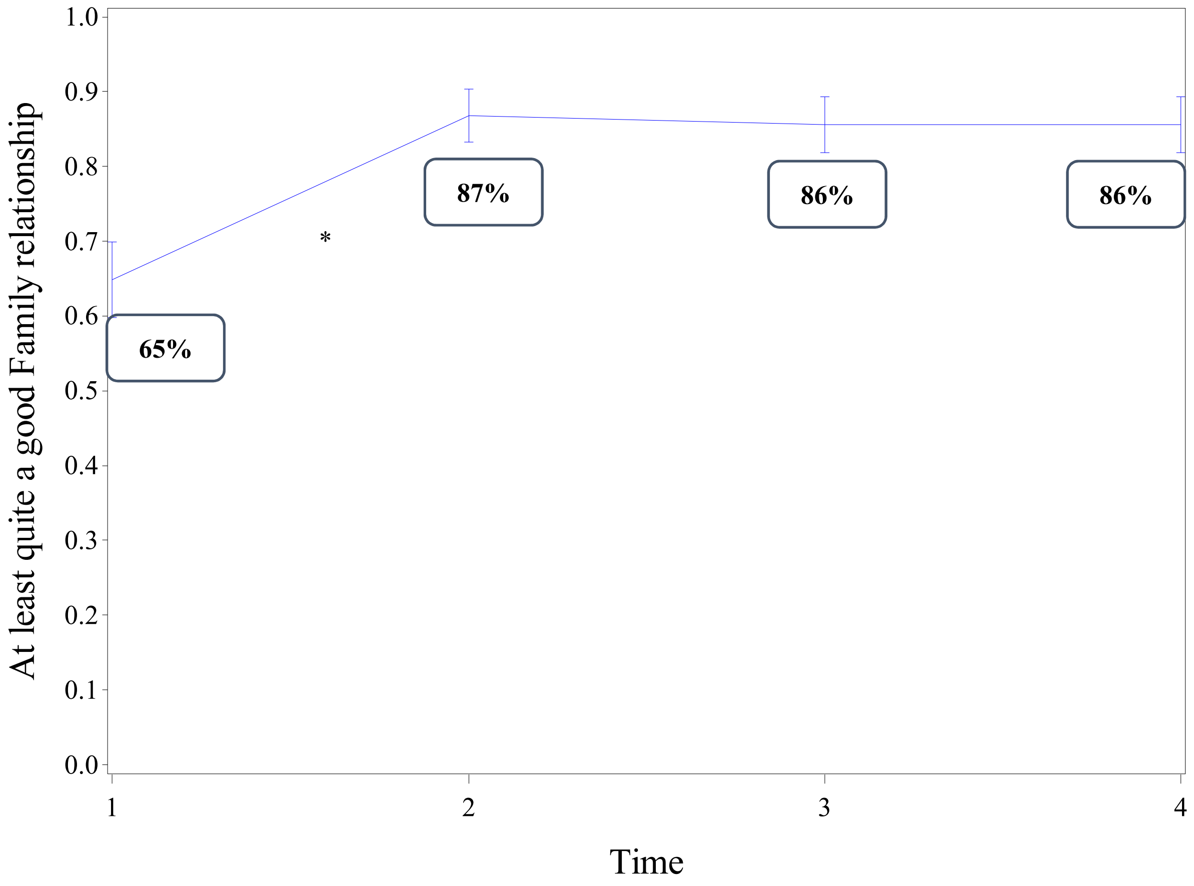

Figure S2a. Change in the proportion of patients who were referred to primary care and whose QoL ratings for family relationship were at least "quite good" for family relationship, at different times: Time 1: at entry into the specialized center (inclusion); Time 2: last visit to the specialized center; Time 3: at entry into primary care; Time 4: last visit to primary care. A significant time effect is indicated by "*".

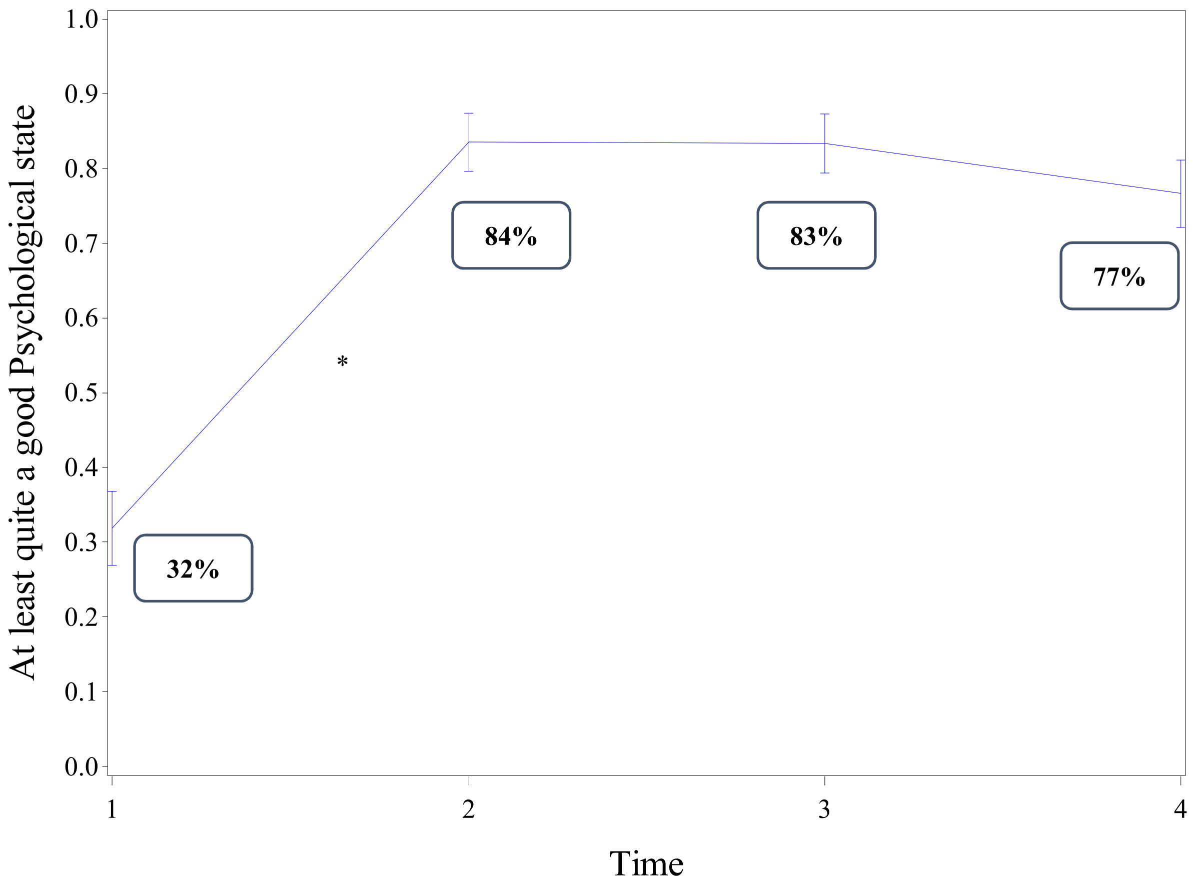

Figure S2b. Change in the proportion of patients who were referred to primary care and whose QoL ratings for psychological state were at least “quite good”, at different times: Time 1: at entry into the specialized center (inclusion); Time 2: last visit to the specialized center; Time 3: at entry into primary care; Time 4: last visit to primary care. A significant time effect is indicated by “*”.

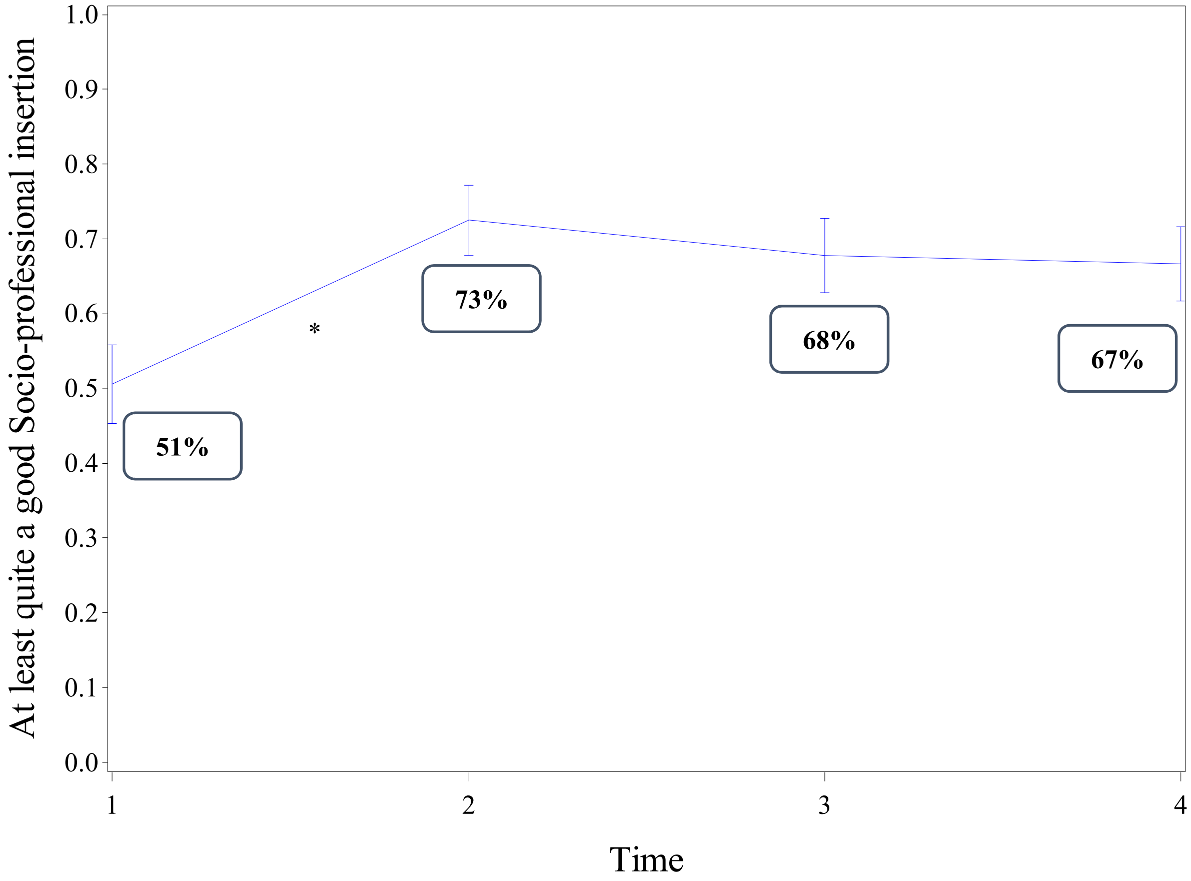

Figure S2c. Change in the proportion of patients who were referred to primary care and whose QoL ratings for socio-professional insertion were at least "quite good", at different times: Time 1: at entry into the specialized center (inclusion); Time 2: last visit to the specialized center; Time 3: at entry into primary care; Time 4: last visit to primary care. A significant time effect is indicated by "*".

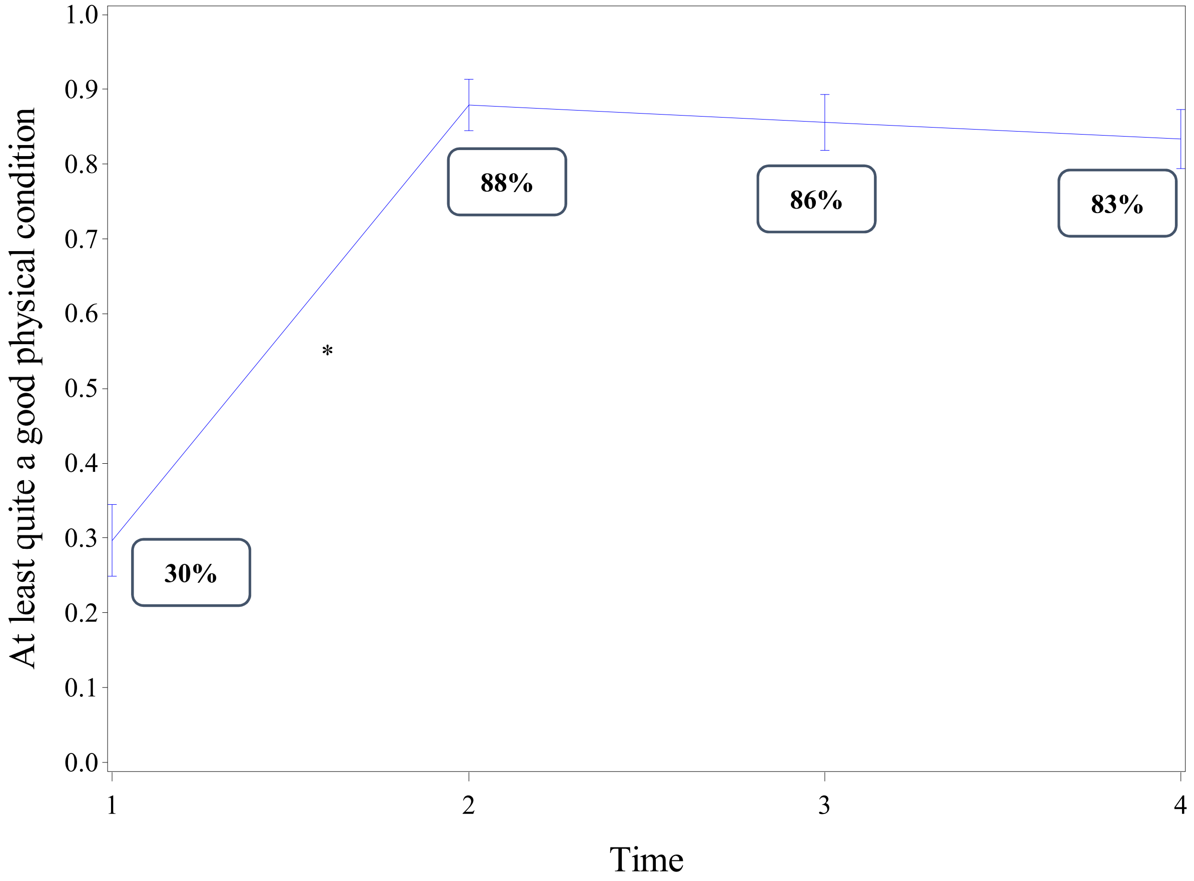

Figure S2d. Change in the proportion of patients who were referred to primary care and whose QoL ratings for physical condition were at least "quite good", at different times: Time 1: at entry into the specialized center (inclusion); Time 2: last visit to the specialized center; Time 3: at entry into primary care; Time 4: last visit to primary care. A significant time effect is indicated by "*".
